# Simultaneous genome-wide association studies of anti-cyclic citrullinated peptide in rheumatoid arthritis using penalized orthogonal-components regression

**DOI:** 10.1186/1753-6561-3-s7-s20

**Published:** 2009-12-15

**Authors:** Yanzhu Lin, Min Zhang, Libo Wang, Vitara Pungpapong, James C Fleet, Dabao Zhang

**Affiliations:** 1 Department of Statistics, Purdue University, West Lafayette, Indiana 47907, USA; 2 Department of Foods and Nutrition, Purdue University, West Lafayette, Indiana 47907, USA

## Abstract

Genome-wide associations between single-nucleotide polymorphisms and clinical traits were simultaneously conducted using penalized orthogonal-components regression. This method was developed to identify the genetic variants controlling phenotypes from a massive number of candidate variants. By investigating the association between all single-nucleotide polymorphisms to the phenotype of antibodies against cyclic citrullinated peptide using the rheumatoid arthritis data provided by Genetic Analysis Workshop 16, we identified genetic regions which may contribute to the pathogenesis of rheumatoid arthritis. Bioinformatic analysis of these genomic regions showed most of them harbor protein-coding gene(s).

## Background

Most of the available statistical approaches for genome-wide association study (GWAS) have focused on studying one single-nucleotide polymorphism (SNP) at a time [1], thereby ignoring the multigenic nature of complex diseases [2,3] and the strong correlation between some SNPs due to linkage disquilibrium (i.e., some SNPs are inherited together in blocks of DNA). As pointed out by Waldron et al. [4], association studies using multiple SNPs have substantial advantages over those based on SNP associations. To capture the correlation between SNPs in regions of low recombination, haplotype-based methods that recognize the existence of linkage disequilibrium between genetic markers have been employed for multi-SNP analyses. However, such methods introduce additional problems, including the need to infer haplotypes, the impact of including (or excluding) rare haplotypes, and the need to define haplotype block boundaries. Even with haplotype-based association methods, high false-positive rates and a lack of reproducibility remain major concerns.

Because hopes remain high for the value of GWAS, other approaches need to be pursued that account for the correlation structure among SNPs. Here we have developed a method for GWAS that incorporates regression of a phenotype simultaneously on all available SNPs, i.e., considering a multiple linear regression,

where **Y **is the phenotypic value, **X**_*j *_counts one of the two alleles at the *j*^th ^SNP, and *β*_*j *_is the additive effect of that allele, *j *= 1,..., *p*. To account for the issues raised by large *p*, such as lack of independence between SNPs due to linkage disequilibrium, we will conduct the GWAS using the penalized orthogonal-components regression (POCRE) [5]. POCRE offers a fast and efficient way to identify significant SNPs simultaneously from a large number of candidates.

## Methods

GWAS usually entails the collection of a massive amount of SNPs (i.e., large *p*) for only a small number of biological individuals (i.e., small *n*). Therefore, identifying the few genetic variants underlying disease risk is equivalent to the task of finding "a very few needles in a haystack", and poses a challenging statistical issue. Zhang et al. [5] recently described the POCRE approach, which sequentially constructs sparsely loaded orthogonal components with proper regularization. They demonstrate this approach works well when fitting regression models with *n *<<*p *data.

Let **Y **be an *n*-dimensional column phenotype vector, and **X **be an *n *× *p *genotype matrix, where *n *and *p *are the number of individuals and number of SNPs, respectively. Further assume both **Y **and **X **are centralized, and accordingly assume *μ *= 0 in Eq. (1). POCRE sequentially constructs orthogonal components , where  and , *k *≥ 2 is iteratively built to be orthogonal to . The loading *ω*_*κ*_, *k *≥ 1, is obtained as *γ*/||*γ*||, which minimizes

where *g*_*λ*_(*γ*) is a penalty function defined by a proper regularization on *γ *with tuning parameter *λ*.

When *g*_*λ*_(*γ*) ≡ 0, the optimal *γ *solving Eq. (2) is proportional to the leading eigenvector of . Zhang et al. [5] employed empirical Bayes thresholding methods proposed by Johnstone and Silverman [6] to introduce proper penalty *g*_*λ*_(*γ*). Such penalty benefits estimating covariance between phenotype and genotypes, and provides adaptively sparse loadings of orthogonal components. The empirical Bayes implementation is also computationally efficient. The tuning parameter *λ *also accounts for possible dependence structure among different SNPs, and 10-fold cross-validation was employed to elicit its optimal values ranging from 0.8 to 1, i.e., considering candidate values *λ *∈ {0.8, 0.82, 0.84, 0.86, 0.88, 0.9, 0.92, 0.94, 0.96, 0.98, 1}.

The sequential construction of the orthogonal components stops when the optimal *γ *solving Eq. (2) is zero, which implies  is hardly correlated to **Y **Then, regressing **Y **on the orthogonal components, i.e., , provides an estimate of *β*_1_,⋯, *β*_*p *_in Eq. (1). Because non-zero loadings in *ω*_*j*_, *j *= 1,2,⋯, are sparse, most of estimated *β*_1_,⋯, *β*_*p *_are therefore zero, reflecting the fact that most SNPs are insignificantly associated or are even completely uncorrelated to the phenotype of interest.

## Results

Using our novel method (POCRE), the rheumatoid arthritis data from Genetic Analysis Workshop 16 were investigated for associations between SNPs and a serum biomarker for rheumatoid arthritis, i.e., antibodies anti-cyclic citrullinated peptide (anti-CCP). In this dataset, only 867 samples were positive for anti-CCP. The data set was preprocessed with the computer program PLINK [7] to control data quality, and with the computer program EIGENSTRAT [8] to control potential population structures. After preprocessing there were 490,613 SNPs remaining for the GWAS. POCRE was applied individually to each chromosome for the simultaneous association of the SNPs in that chromosome with the anti-CCP phenotype. The effects of the 10 principal components constructed by EIGENSTRAT were considered as covariates for the POCRE. Nonzero effects of SNPs were reported on seven chromosomes where the positive  indicates that the minor allele will increase the level of anti-CCP (Table 1).

**Table 1:** SNPs identified with nonzero coefficients in Eq. (1)

SNP		Chromosome	SNP Location (bp)	Gene	Gene Location (bp)
rs233492	149.7514	6p23	14,910,666	*JARID2*^a^	14,754,745-14,754,848
rs17068819	109.0095	6q21	108,246,263	*SCML4*	108,130,060-108,252,214
rs922898	56.7294	6q25.1	149,155,750	*UST*	149,110,165-149,439,818
rs6929401	83.7685	6q25.1	149,158,549	*UST*	149,110,165-149,439,818
rs17087579	187.2452	6q25.1	149,886,810	*PPIL4*	149,867,324-149,908,864
rs11760836	358.7046	7p21.2	14,903,193	*DGKB*	14,151,199-14,909,359
rs11029744	300.2939	11p14.2	26,909,033	*AC016450.10*	26,972,204-26,975,206
rs10861038	63.7626	12q23.3	102,455,524	*STAB2*^a^	102,505,181-102,684,635
rs10507167	117.5381	12q23.3	102,490,212	*STAB2*^a^	102,505,181-102,684,635
rs17055893	265.3688	13q13.3	36,961,628	*POSTN*^a^	37,034,779-37,070,874
rs2322047	324.3976	17p12	11,556,512	*DNAH9*	11,442,473-11,813,856
rs9305833	102.7568	21q21.1	18,238,917	*CHODL*	18,195,451-18,561,561

^a^SNP does not reside in any gene. The nearest gene is shown.

Of the 12 SNPs identified to be associated with anti-CCP level, 5 SNPs are from chromosome 6, 2 SNPs from chromosome 12, and 1 SNP each is from chromosomes 7, 11, 13, 17, and 21, respectively. The location of each significant SNP was mapped to the human genome using the Ensembl database http://www.ensembl.org. Based on this analysis, eight of the SNPs were found to reside in seven genes (Table 1). For the other four SNPs, the nearest neighboring genes are listed in the table. None of these genes we identified have previously been linked to rheumatoid arthritis, but several of them encode proteins whose biological roles may be involved in the pathogenesis of this disease. For example, two SNPs (rs922898, rs6929401) that reside in the uronyl-2-sulfotransferase (*UST*) gene were identified by our method. *UST *is involved in the chondroitin 3 sulfate and glycan structure biosynthesis pathways and could contribute to optimal cartilage development or repair. *PPIL4 *is a member of cyclophilin family, a group of proteins crucial for protein folding and immunosuppression by cyclosporin A (CsA) [9]. Polymorphisms in this gene may influence development of inflammation during rheumatoid arthritis or the response of individuals with rheumatoid arthritis to treatment. *DGKB *encodes diacylglycerol kinase and participates in intracellular signalling processes via several pathways including the protein kinase C pathway [10]. Alteration in intracellular signalling could negatively influence inflammatory processes. Finally, *CHODL *encodes a type I transmembrane protein including a single carbohydrate recognition domain for the C-type lectins that can worsen inflammation [11].

## Discussion

In addition to its diagnostic value for the general risk of rheumatoid arthritis, a high anti-CCP level is associated with a high risk of developing joint damage during the disease. As a result, an anti-CCP-test has been employed to monitor the progression of the disease. With our novel approach for GWAS, we identified several candidate SNPs associated with the level of anti-CCP in rheumatoid arthritis patients. Among the candidate genes identified, only *PPIL4 *is functionally related to the immune system. Further investigation will be necessary to define the potential roles that the other gene products play in rheumatoid arthritis. Due to the small sample size available for this study (867) and the large total number of SNPs measured (490,613), we expect some of the associations we identified to constitute false positives. Determining false detection rates will require development of additional procedures.

## Conclusion

Our analyses using the newly developed method POCRE indicate that the genomic region 6q25.1 may harbor genes associated with anti-CCP level in rheumatoid arthritis patients. Further investigation is necessary to confirm this observation.

## List of abbreviations used

anti-CCP: Anti-cyclic citrullinated peptide; GWAS: Genome-wide association study; POCRE: Penalized orthogonal-components regression; SNP: Single-nucleotide polymorphism.

## Authors' contributions

YL and MZ designed the study, and YL performed the statistical analysis. MZ and DZ both conceived the study, and drafted the manuscript. LW and VP participated in the design of the study and preprocessing of the data. JCF participated in interpreting the statistical analysis results, reviewing and editing the manuscript. All authors read and approved the final manuscript.
